# The multifaceted roles of extracellular vesicles for therapeutic intervention with non-Hodgkin lymphoma

**DOI:** 10.20517/evcna.2024.07

**Published:** 2024-06-26

**Authors:** Arthur A. Lee, Andrew K. Godwin, Haitham Abdelhakim

**Affiliations:** ^1^Department of Pathology and Laboratory Medicine, University of Kansas Medical Center, Kansas City, KS 66160, USA.; ^2^Bioengineering Program, The University of Kansas, Lawrence, KS 64111, USA.; ^3^Kansas Institute for Precision Medicine, University of Kansas Medical Center, Kansas City, KS 66160, USA.; ^4^The University of Kansas Cancer Center, University of Kansas Medical Center, Kansas City, KS 66160, USA.; ^5^Division of Hematologic Malignancies and Cellular Therapeutics, University of Kansas Medical Center, Kansas City, KS 66160, USA.

**Keywords:** Extracellular vesicles, non-Hodgkin lymphoma (NHL), diffuse large B-cell lymphoma (DLBCL), R-CHOP, autologous stem cell transplantation (ASCT), chimeric antigen receptor T-cell (CAR-T) therapy, biomarkers, microfluidic devices

## Abstract

Extracellular vesicles (EVs) contribute to the development of cancer in various ways. Non-Hodgkin lymphoma (NHL) is a cancer of mature lymphocytes and the most common hematological malignancy globally. The most common form of NHL, diffuse large B-cell lymphoma (DLBCL), is primarily treated with chemotherapy, autologous stem cell transplantation (ASCT), and/or chimeric antigen receptor T-cell (CAR-T) therapy. With NHL disease progression and its treatment, extracellular vesicles play remarkable roles in influencing outcomes. This finding can be utilized for therapeutic intervention to improve patient outcomes for NHL. This review focuses on the multifaceted roles of EVs with NHL and its potential for guiding patient care.

## INTRODUCTION

Extracellular vesicles (EVs) have been widely demonstrated as playing significant roles in the development of cancer^[1-3]^ and are emerging as a new paradigm of liquid biopsy for non-invasive cancer diagnosis and monitoring^[4-11]^. The International Society for Extracellular Vesicles (ISEV) defines EVs as “particles naturally released from the cell that are delimited by a lipid bilayer and cannot replicate”^[12]^. EVs are composed of three general subclasses: ectosomes, exosomes, and apoptotic bodies^[12-14]^. These subclasses reflect the differing processes for the biogenesis of EVs from cells. Ectosomes, also known as microvesicles (MVs), develop through outward budding of the plasma membrane that pinches off to release contents of the cells as cargo in the EVs^[13,14]^. Exosomes or small EVs (sEVs) form via the endosomal sorting complex required for transport (ESCRT) pathway^[13,14]^. Inward budding of the cell membrane results in intraluminal vesicles (ILVs) within a multivesicular body (MVB)^[13,14]^. As the MVB rejoins the cell membranes, these ILVs are released from the cell as exosomes^[13,14]^. Apoptotic bodies (ApoBDs) are generated from fragments of the cell during apoptosis. Ectosomes range in size from 100-1,000 nm^[14]^, while exosomes are generally 30-150 nm and ApoBDs are 50-5,000 nm^[14]^.

Extracellular vesicles vary in their functions to support cancer pathogenesis^[2]^. Hanahan and Weinberg pointed out the six hallmarks of cancer to be sustaining proliferation, evading growth suppression, enabling replicative immortality, resisting cell death, inducing angiogenesis, and activating invasion and metastasis^[15]^. Much research has implicated EVs in contributing to all these hallmarks in the last twenty years. Scenarios for cancer cell EV release include cellular fragmentation following cell death as ApoBDs, external budding from cancer cells as ectosomes, and formation of small vesicles through the ESCRT pathway as exosomes^[1-3,16]^. This shedding of EVs into extracellular space induces prominent cell-to-cell signaling as EVs are received by other cells through endocytosis^[1-3,16]^. EVs have been widely demonstrated to carry cargos of DNA, RNA, peptides, proteins, and metabolites^[1-14,16-22]^. The transfer of EV cargo to other cells for cancer progression occurs in both the immediate vicinity of the tumor microenvironment and more distal sites through extravasation for metastasis and angiogenesis^[1-11,20-22]^. By transferring to additional cancer cells, EVs sustain proliferation primarily through activation of signal transduction in the phosphatidylinositol 3-kinase / protein kinase B (PI3K/AKT) and the mitogen-activated protein kinase / extracellular signal-regulated kinase (MAPK/ERK) pathways^[1-3]^. To evade growth suppression, EVs from cancer cells disseminate mutated p53, transfer microRNAs (miRNAs) to silence tumor suppressors, and discard tumor suppressor miRNAs and membrane-associated protein phosphatase and tensin homolog (PTEN) during EV release^[1,23,24]^. Like their originating cancer cells, they also avoid immune destruction through the downregulation of immune cells. EVs express PD-L1 and CTLA-4, as well as CD39 and CD73, to convert adenosine from ATP, thereby suppressing T cell activity^[2,3,25]^. EVs also carry NKG2D ligands to act as targets for natural killer (NK) cells instead of their originating cancer cells^[3,25]^. To enable replicative immortality and escape senescence, cancer cells release EVs to transport telomeric non-coding RNAs (ncRNAs) for extending telomeres in neighboring cells^[26-28]^. EVs help cancer cells resist death by modifying BCL2 protein expression, communicating factors to help neighboring cancer cells adapt, discarding chemotherapy agents such as cisplatin from the cell, and acting as decoys to monoclonal antibodies such as trastuzumab^[29-33]^. For cancer angiogenesis, EVs secrete vascular endothelial growth factor (VEGF) and disseminate epidermal growth factor receptor (EGFR) molecules for activation within the tumor microenvironment^[2,3,34]^. With their ability to migrate and extravasate, cancer EVs invade distal sites in the body to form a pre-metastatic niche^[1-3]^. They facilitate metastasis by secreting factors, such as matrix metalloproteinases (MMPs), a disintegrin and metalloproteinases (ADAMs), and miRNAs, such as miR-19a to downregulate PTEN expression^[1-3]^. These many roles of EVs in cancer pathogenesis provide ample opportunities for clinical data^[4-11,17-20]^.

**Figure 1:**
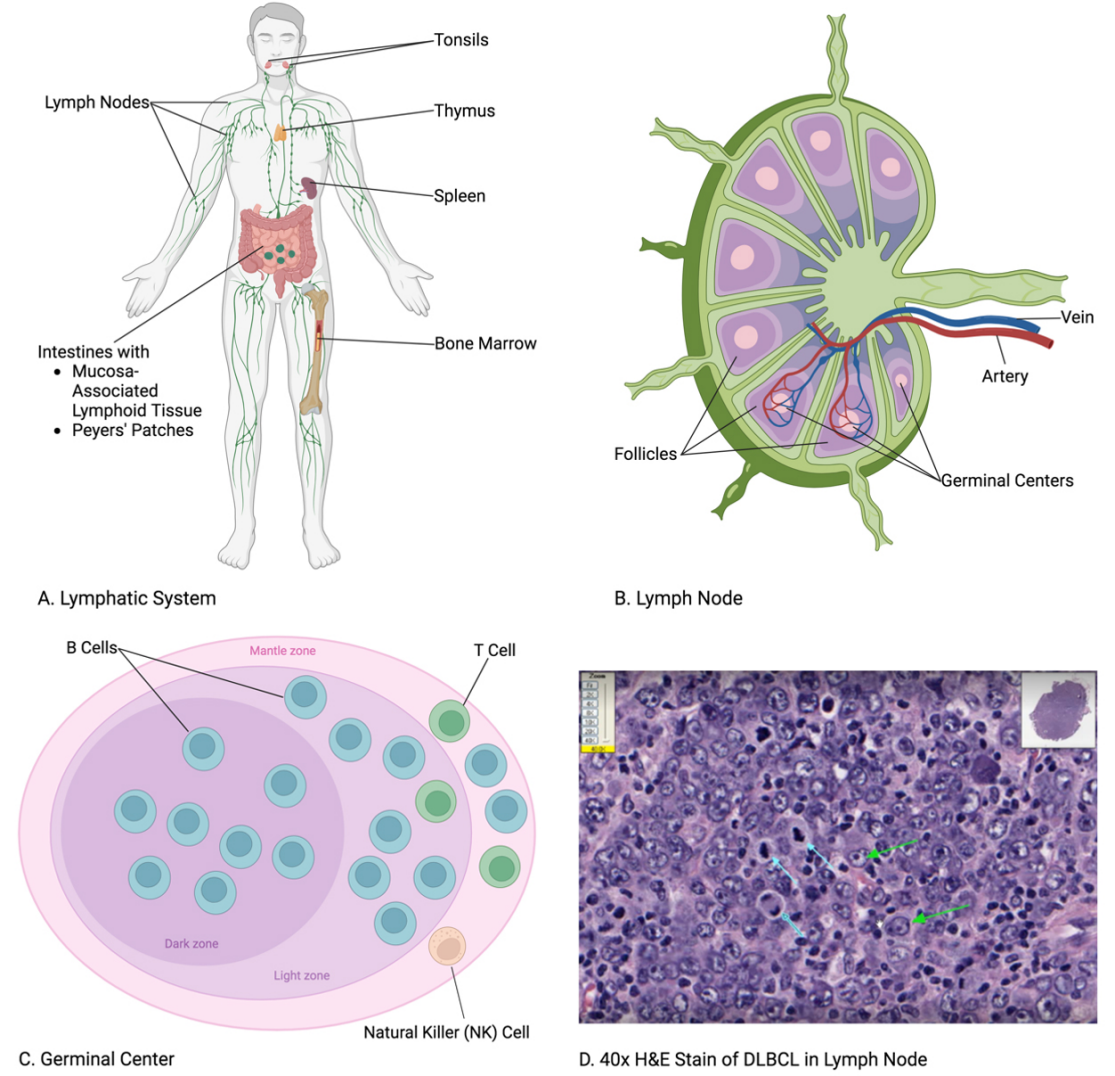
Non-Hodgkin lymphoma (NHL) refers to cancers that develop through B, T, and natural killer (NK) cells in the lymphatic system^[35]^. (A) Lymphatic System: Tissues and organs susceptible to NHL include the lymph nodes, tonsils, thymus, spleen, bone marrow, and intestines via the mucosa-associated lymphoid tissue (MALT) and Peyers’ patches^[36]^. (B) Lymph Node: Blood and lymph interface for lymphocyte recirculation^[37]^. (C) Germinal Center: B cells account for about 85% of NHL cases in the United States, with T cells less than 15% and NK cells less than 1%^[38]^. (D) 40x H&E Stain of DLBCL in Lymph Node: Diffuse large B-cell lymphoma (DLBCL), the most common form of NHL, is identified in a hematoxylin and eosin stain by the neoplastic B cells with large vesicular nuclei (green arrows) and mitotic bodies (blue arrows) among the diffuse proliferation of lymphocytes^[39]^ (the images in the figure are not drawn to exact scale).

Growing evidence indicates EVs’ significant influence on non-Hodgkin lymphoma (NHL) with disease progression and its treatment. NHL is a type of cancer that originates in the lymphatic system, as shown in Figure 1. As the most common hematological malignancy globally, NHL refers to cancers of mature lymphocytes such as B and T cells, excluding Hodgkin lymphoma^[35,40,41]^. As 90% of all lymphomas, NHL is the 11th most commonly diagnosed cancer globally and the 8th most in the United States, with 4% of all new cancer cases^[41-43]^. The most common form of NHL is diffuse large B-cell lymphoma (DLBCL), accounting for approximately 25%-30% of all NHL cases worldwide^[44]^. Immunoglobulin gene recombination and somatic hypermutation for class switching and affinity maturation of the mature immunoglobulin causes B cells to be susceptible to oncogenesis through these genetic events and the most common cell of origin for lymphoma^[35]^. B-cell lymphomas result from these genetic aberrations in different stages of B-cell development, primarily in the lymph nodes and the spleen^[45-47]^. Due to their direct interaction with NHL disease progression, EVs are increasingly demonstrated as biomarkers for NHL outcomes^[3,41,48]^.

The standard first-line therapy for DLBCL is a combination chemotherapy, R-CHOP [rituximab, cyclophosphamide, doxorubicin hydrochloride (hydroxydaunomycin), vincristine sulfate (oncovin), and prednisone]^[17,35,49]^. This cures about 60%-70% of DLBCL patients^[17,35,49,50]^. For patients with refractory or relapsed DLBCL, the ensuing lines of therapy include autologous stem cell transplantation (ASCT) or chimeric antigen receptor T-cell (CAR-T) therapy^[49,50]^. For NHL response with these therapies, EVs have been indicated as biomarkers, influencing outcomes of treatment, and a treatment in itself. Here, we will explore these effects and the potential for EVs to improve NHL patient care.

## BIOMARKER FEATURES OF EVS FOR NHL TREATMENT

**Figure 2:**
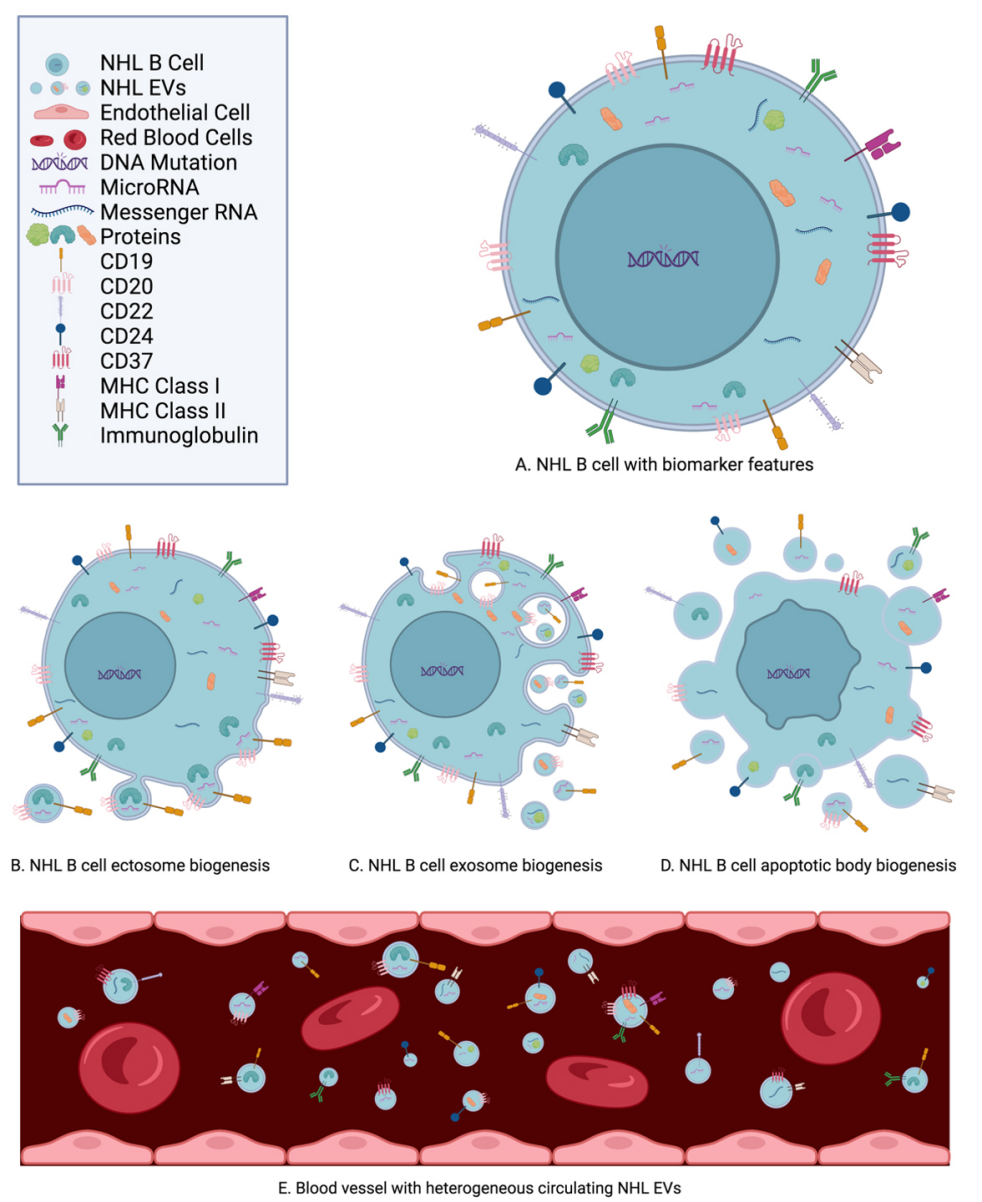
Features of NHL EVs as biomarkers in blood include exo-microRNA, exo-messenger RNA, and exo-proteins, such as CD19, CD20, CD22, CD24, CD37, MHC class I molecules, MHC class II molecules, and immunoglobulins^[17,41,48,51,55,56]^. (A) NHL B cell with biomarker features: Most forms of non-Hodgkin B cell lymphoma originate in the lymph nodes during B cell maturation^[45-47]^. (B) NHL B cell ectosome biogenesis: Outward budding pinches off to release contents of the cell as cargo packaged within the cell’s membrane and surface proteins as EV microvesicles^[13,14,41]^. (C) NHL B cell exosome biogenesis: Through the endosomal sorting complexes required for transport (ESCRT) pathway, cell membrane surface proteins (such as CD19 or CD20) invaginate into the cell. Within multivesicular bodies, intraluminal vesicles form with these surface membrane proteins to carry contents of the NHL B cells, such as RNA and proteins. These vesicles are expelled from the cell as EV exosomes^[13,14,41]^. (D) NHL B cell apoptotic body biogenesis: Apoptosis fragments into newly formed EV apoptotic bodies, with the membrane and its surface proteins enveloping cell contents of RNA and proteins^[13,14,41]^. (E) Blood vessel with heterogeneous circulating NHL EVs: Heterogeneous subpopulations of NHL EVs are released into environs of the NHL cells and into the bloodstream. Varying in size, they carry diverse contents internally with various RNA and proteins and externally with differing antigens, MHC class molecules, and immunoglobulins^[17,41,48,51,55,56]^ (images in the figure are not drawn to exact scale).

In comparison to circulating tumor cells and cell-free DNA, EVs are relatively more abundant in the blood, with a distribution half-life of 19.9 min and an elimination half-life of 184.5 min in mice^[17,48,51]^. EVs retain the phenotypic characteristics of NHL cells, providing ample opportunities as biomarkers^[17,41,48,52]^. Biomarkers are commonly categorized as either diagnostic, prognostic, or predictive. Diagnostic biomarkers indicate the presence or absence of the disease^[52]^. Prognostic biomarkers provide information about disease outcomes^[53,54]^. Predictive biomarkers supply information about treatment benefits^[53,54]^. Based on reliable biomarkers, medical personnel can adjust NHL treatment to achieve optimal outcomes. Distinguishing features of EVs as biomarkers include their RNA, proteomes, antigen expression, and immunoglobulin expression^[17,41,48,51,55,56]^. These features can be classified as diagnostic, prognostic, or predictive biomarkers for NHL, which are illustrated in Figure 2.

### RNA

Some of the most compelling evidence for biomarkers for NHL comes from RNA in EVs, specifically microRNA (miRNA) and messenger RNA (mRNA). Cells communicate with EVs to exchange RNA, with miRNA as the most numerous cargo molecules in EVs^[56]^. MicroRNA are single-stranded, non-coding RNA molecules of approximately 19-22 nucleotides long and act as regulators of protein biosynthesis^[56,57]^. Ribonucleoprotein complexes possibly help the stability of miRNA in plasma EVs^[58]^. Messenger RNA, which are single-stranded molecules for synthesizing proteins by ribosomes, are usually smaller than 1 kb in EVs^[57,59]^. They present novel proteins to recipient cells, even translating within one hour after EV uptake between co-cultures of glioblastoma and HEK293T cells^[60]^.

Cao *et al.* examined which miRNAs were most pertinent for DLBCL from serum EVs isolated with ExoQuick Exosome Precipitation Solution by System Biosciences^[61]^. From 24 DLBCL patients *vs.* 24 healthy controls, three circulating EV miRNAs, miR-379-5p, miR-135a-3p, and miR-4476, had higher expression while two, miR-483-3p and miR-451a, had lower expression in DLBCL patients with fold changes greater than 1.5 and *P*-values less than 0.05^[61]^. In a larger cohort of 99 DLBCL patients *vs.* 65 healthy controls, this 5-miRNA signature panel was validated for diagnosis with an area under the receiver operating characteristic curve (AUC) of 0.90, higher than each of the miRNAs independently, along with a sensitivity of 0.83 and a specificity of 0.85^[61]^. When tested with another 29 non-DLBCL lymphoma subtype cases, miR-379-5p, miR-135a-3p, miR-4476, and miR-451a still showed significant differential expression between DLBCL *vs.* non-DLBCL lymphoma along with DLBCL *vs.* healthy, but not non-DLBCL lymphoma *vs.* healthy. Only miR451a demonstrated significant diagnostic differential expression between all three cases, with non-DLBCL lymphoma as the lowest, DLBCL in the middle, and healthy as the highest^[61]^. MiR451a was also investigated for its prognostic value and shown to have higher rates of both progression-free survival (PFS) and overall survival (OS) at higher expression^[61]^. With the exoEasy Maxi Kit, a different study enriched EVs from plasma samples of 42 DLBCL patients and 31 healthy controls^[62]^. From these plasma EVs, miR-107 expression was downregulated in DLBCL patients. MiR-107 expression is affiliated with cell apoptosis and tumor suppression, indicating its potential as a diagnostic marker^[62]^.

In another study with serum EVs from 89 DLBCL patients and 49 healthy controls, Xiao *et al.* also used ExoQuick by System Biosciences to demonstrate miR451a expression as lower in DLBCL patients than healthy controls^[63]^. They found lower levels of miR451a affiliated with worse responses (none *vs.* partial *vs.* complete) to R-CHOP treatment, indicating its capacity as a predictive biomarker in addition to diagnosis and prognosis^[63]^. Their AUC for EV miR451a as a predictive biomarker for response to R-CHOP treatment was 0.8038^[63]^. With ExoQuick enrichment of serum EVs from 116 DLBCL patients, Feng *et al.* found miR-99a-5p and miR-125b-5p to be significantly upregulated and associated with shorter progression-free survival time in response to R-CHOP^[64]^. With Exo-spin Purification by Cell Guidance Systems, EV miR-155 was found to be significantly higher in the plasma of refractory/relapsed DLBCL patients *vs.* DLBCL patients who responded well to R-CHOP^[65]^. Yazdanparast *et al.* summarized this and other results with methods of EV enrichment in a systematic review of twelve biomarker research articles^[66]^. They found EV miRNA in DLBCL as diagnostic in nine articles, prognostic in three articles, and predictive in four articles^[66]^.

Provencio *et al.* conducted a study with messenger RNA of *C-MYC, BCL-XL, BCL-6, NF-kB, PTEN,* and *AKT* from EVs to investigate prognostic and predictive values for NHL, since these six genes contribute to deregulated pathways in cancer^[67]^. The mRNA in EVs was obtained by differential centrifugation of plasma from healthy donors or NHL patients^[67]^. As a potential diagnostic tool, *BCL-*6 EV mRNA was detected in more NHL patient plasma samples while *PTEN* EV mRNA was present in more healthy donor plasma samples^[67]^. For prognosis, the pretreatment presence of *BCL-6* or *C-MYC* EV mRNA was associated with worse progression-free survival (PFS) and worse overall survival (OS)^[67]^. The absence of *PTEN* EV mRNA indicated worse PFS^[67]^. As a predictive biomarker to rituximab-based treatment, the presence of *AKT* EV mRNA was associated with no response and worse PFS^[67]^. The presence of *C-MYC* EV mRNA was also a significant predictor for lack of complete response (CR) to R-CHOP^[67]^.

Another study by Bang *et al.* analyzed mRNA from EVs ultracentrifuged from NHL patient serum^[68]^. Thirty-three NHL patients each had a different subtype, DLBCL (*n* = 17), intravascular B-cell lymphoma (IVL, *n* = 1), primary mediastinal large B-cell lymphoma (PMBL, *n* = 4), follicular lymphoma (FL, *n* = 3), mantle cell lymphoma (MCL, *n* = 3), or extranodal NK/T-cell lymphoma (ENKTL, *n* = 5), but only enough EV mRNA could be extracted for sequencing from 26 NHL patients (DLBCL: 13/17, IVL: 0/1, PMBL: 4/4, MCL: 3/3, FL: 2/3, ENKTL: 4/5)^[68]^. Among 25 newly diagnosed NHL patients, NHL patients with enough detectable EV mRNA had worse PFS than those without it^[68]^. Though with a low number of patients, the EV mRNA expression profiles, including *MYC*, *BCL-2*, *BCL-6*, and *CCND1*, were consistent with each of their respective NHL subtypes. This also applied to the cell of origin with activated B cell (ABC) or germinal center B cell (GCB) type for DLBCL and PBML. Furthermore, their EV mRNA showed a closer association than circulating tumor DNA (ctDNA) with NHL relapse after treatment^[68]^. Additionally, with EVs from ultracentrifugation, Rutherford *et al.* found EV mRNA from DLBCL cell lines had mutations, indicating the cell of origin for the disease^[17,69]^. Distinct outcomes and different responses to R-CHOP were demonstrated between genetic aberrations within DLBCL subtypes of ABC and GCB as the cell of origin^[47,48,70]^. This non-invasive biomarker could improve diagnosis, prognosis, and predictions for R-CHOP outcomes.

### Proteomes

Proteins and peptides within EVs also present information as diagnostic, prognostic, and predictive biomarkers. Though NHL was not in this particular study, EV proteomic analysis detected and determined different cancer types, including pancreatic, lung, melanoma, neuroblastoma, and osteosarcoma cancers^[71]^. Two separate studies used proteomic profiling to determine the cell of origin for DLBCL patients using formalin-fixed, paraffin-embedded (FFPE) tissue sections^[72,73]^. Instead of FFPE tissues, EVs enriched from sucrose cushion ultracentrifugation were recently used to demonstrate similar proteomic profiling from DLBCL patients^[74,75]^. With state-of-the-art mass spectrometry, EV proteomic analysis of patient DLBCL cell lines distinguished between ABC and GCB as the cell of origin^[74]^. These same methods of enrichment and analysis were applied to plasma EVs from 32 DLBCL patients treated with R-CHOP *vs.* 15 age-matched healthy donors to detect cancer and indicate outcomes^[75]^. With nanoparticle tracking analysis (NTA) applied before treatment, DLBCL patients were found to have a higher number of overall particles and a greater variance of size in the EV size range, as compared to healthy donors^[75]^. A qualitative comparison of the EV proteome between the two groups identified more unique proteins, with a more diverse proteome and richness in isoforms of these unique proteins, for DLBCL patients compared to healthy donors^[75]^. Additionally, functional enrichment analysis with the Kyoto Encyclopedia of Genes and Genomes (KEGG) showed DLBCL EV proteins to be more enriched in proteasomes, infection-related functions, antigen presentation, and glycolysis and gluconeogenesis functions^[75]^. More differentially expressed proteins were also revealed from DLBCL EVs for lupus-related systemic inflammation, platelet activation, regulation of glycoprotein metabolic processes, and GTPase activity^[75]^. Higher expression of immunoglobulin lambda constant 1 (IGLC1), immunoglobulin lambda-like polypeptide 5 (IGLL5), proteasome subunit beta type-2 (PSMB2), and coronin-1a (CORO1a) were indicated in Kaplan-Meier plots to have a lower survival probability among these R-CHOP-treated DLBCL patients^[75]^. These same four protein markers were also associated with poor prognosis in other cancers^[75]^.

### Antigen expression

NHL EVs also retain the phenotypes of NHL cells through antigen expression^[41]^. NHL EVs express generic EV proteins such as intercellular adhesion molecule 1 (ICAM-1), ESCRT proteins (Alix, TSG101), tetraspanins (CD63, CD81), and heat shock proteins (HSP70, HSP90)^[16,41,69,76-78]^. Characteristic of NHL cells, B-cell lymphoma EVs also express CD19, CD20, CD22, CD24, CD37, and major histocompatibility complex (MHC) class I and II molecules^[16,41,69,76,77]^. As discovered with flow cytometry on ultracentrifuged EVs, higher serum levels of CD19+ and CD20+ EVs from 33 B-cell lymphoma patients than 28 healthy donors indicate potential as a diagnostic biomarker^[79]^.

### Immunoglobulin+ and VDJ Recombination

Given that the majority of NHL cases originate from B cell development during immunoglobulin (Ig) maturation, V(D)J recombination for immunoglobulins provides an opportunity to find specific sequences unique to the cancer and the patient^[35,37]^. The variable regions on immunoglobulins undergo changes during somatic hypermutation for affinity maturation to specific antigens^[37]^. Immunoglobulin expression on B cells is expected, yet it has also been found on numerous types of cancer cells^[80]^. This phenomenon occurs with isotypes IgG, IgA, IgM, as well as Ig and Ig^[80]^. Recently, immunoglobulin M expression on EVs from murine B lymphocyte cells has been confirmed to bind to antigens and lead to cell uptake^[55]^. Kurtz *et al.* investigated high-throughput sequencing of immunoglobulin genes (IgHTS) for monitoring DLBCL with 75 patients undergoing treatment, most of them with R-CHOP^[81]^. From tumor biopsy, clonotypic immunoglobulin rearrangement was identified in 57 of the patients, with heavy-chain VDJ rearrangement (*IGH-VDJ*) in 39, heavy-chain DJ rearrangement (*IGH-DJ*) in 23, and κ light-chain VJ rearrangement (*IGK*) in 25^[81]^. Between the two types of circulating DNA for these 57 patients, plasma cell-free DNA correlated better than circulating leukocytes with positron emission tomography combined with computed tomography (PET/CT) for detecting metabolic tumor volume (MTV) as a measurable residual disease (MRD)^[81]^. At the time of relapse for 11 patients, plasma cell-free DNA detected MRD at 100% *vs.* circulating leukocytes at 30% (*P* = 0.001)^[81]^. As compared to PET/CT scans for 25 patients, plasma IgHTS showed better specificity at 100% *vs.* 56% (*P* < 0.001)^[81]^. More recently, IgHTS was used to identify (MRD) in DLBCL patients undergoing autologous stem cell transplantation (ASCT)^[82]^. Among 98 patients who had apheresis stem cell (ASC) samples taken and 60 with post-ASCT surveillance, the 5-year PFS was 48%. MRD was detected in 23% of ASC samples and associated with a worse PFS (13% *vs.* 53%) and OS (52% *vs.* 68%)^[82]^. As products of V(D)J rearrangements of immunoglobulin genes, Khodadoust *et al.* “identified a total of 11 immunoglobulin derived neoantigens presented by MHC I from 4 of 7 FL/DLBCL tumors and both CLL samples”^[83]^. From EVs isolated with sucrose ultracentrifugation from plasma, flow cytometry results showed greater EV IgG+ expression in pancreatic ductal adenocarcinoma (PDAC) patients as compared to healthy controls^[84]^. Additionally, lowering EV IgG+ levels post-treatment indicated a better response to chemotherapy for metastatic PDAC patients (AUC = 0.8311, *P* = 0.0020)^[84]^. Given their preservation of NHL phenotypes and roles in cell-to-cell communication, this aspect of EVs could also be utilized for monitoring the NHL disease and patient response to treatment^[41,78,81-84]^. However, no studies to our knowledge have examined the presence of immunoglobulins on NHL EVs for MRD.

## THERAPEUTIC FEATURES OF EVS WITH NHL TREATMENT

**Figure 3:**
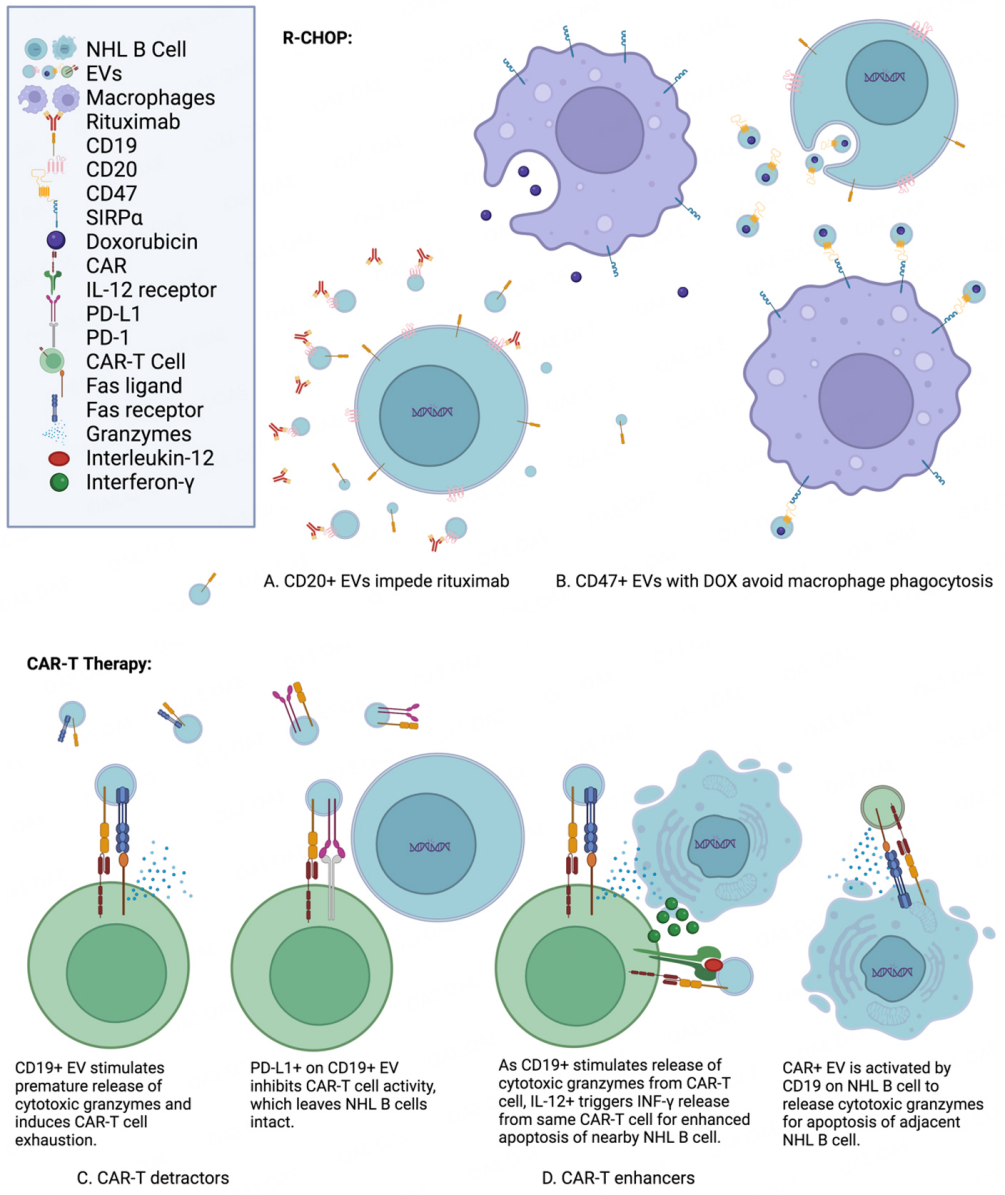
Features of EVs influencing NHL treatments include CD19, CD20, CD47, interleukin-12 (IL-12), programmed death-ligand 1 (PD-L1), doxorubicin, and chimeric antigen receptors (CARs)^[41,76,85-98]^. (A) CD20+ EVs impede rituximab: CD20 antigens on EVs from NHL B cells act as a sink for rituximab. Instead of interacting with CD20 antigens on NHL B cells for antibody-dependent cellular cytotoxicity and complement-dependent cytotoxicity, the monoclonal antibodies are intercepted by CD20+ EVs for a reduction in rituximab efficacy^[41,76,85]^. (B) CD47+ EVs with DOX avoid macrophage phagocytosis: CD47 is ubiquitously expressed on the surface of membranes and signals macrophages not to initiate phagocytosis. It interacts with the signal regulatory protein alpha (SIRP) on the surface of macrophages for inhibitory downstream signaling of phagocytosis^[86]^. Doxorubicin (DOX) enclosed within EVs has better uptake and potency than free DOX^[87]^. (C) CAR-T detractors: CD19 on EVs may stimulate CAR-T cells without any NHL B cells in the vicinity, leading to premature CAR-T cell exhaustion and reduced efficacy^[88]^. PD-L1 on EVs inhibits CAR-T cell activity and diminishes apoptosis of NHL B cells by reducing efficacy of CAR-T cells^[89,90]^. (D) CAR-T enhancers: CD19 on EVs stimulates the release of cytotoxic granzymes from CAR-T cells for apoptosis of nearby NHL B cells^[91]^. Interleukin-12 on the surface of EVs elicits interferon gamma cytokines from the same CAR-T cells for enhanced apoptosis of NHL B cells^[92]^. EVs from CAR-T cells also have the chimeric antigen receptor and the Fas ligand intact from CAR-T cells^[93]^. This enables CAR EVs to induce apoptosis in NHL B cells as well^[94-98]^ (images in the figure are not drawn to exact scale).

EVs show remarkable influences on outcomes for NHL therapies. Their capabilities can be categorized as detractors, enhancers, and potentially new therapeutics. Detractors impede the efficacy of NHL therapies. Enhancers improve the effectiveness of NHL treatments and may possibly be adjuvants. EVs as a new therapeutic in itself are also explored. These categories for EVs are considered with regard to two of the main NHL therapies, R-CHOP and CAR-T therapy. These therapeutic features of EVs for influencing NHL treatments are illustrated in Figure 3.

### R-CHOP

As the first-line therapy and standard-of-care for DLBCL, R-CHOP may be the most imperative for patient recovery to succeed, yet it is susceptible to EV influence^[41,76,85]^. In addition to several chemotherapy medications and the corticosteroid prednisone, R-CHOP includes rituximab as part of its therapeutic regimen^[41,49]^. Rituximab is a monoclonal antibody that targets the CD20 antigen on B cells for antibody-dependent cellular cytotoxicity^[41,49]^. Unfortunately, CD20+ NHL EVs have been demonstrated to reduce rituximab cytolysis of NHL cells through on-target/off-tumor effects and act as a detractor to R-CHOP efficacy^[41,76,85]^. Hence, the evacuation of NHL EVs through dialysis could improve the success of R-CHOP therapy^[41,99]^.

Conversely, EVs are being developed as nanoparticle carriers for chemotherapy^[1,14,78,100-102]^. EVs retain several advantages for drug delivery. Because EVs are integral for cell-to-cell communication, they are naturally apt for cell uptake^[78]^. Their lipid membrane bilayer protects their cargo from degradation^[16]^. EVs are prone to longer circulation due to protection from phagocytosis by CD47 expression on their membrane surface^[102]^. In addition, they have low immunogenicity when administered autologously, can be enhanced for target cell-specificity, and demonstrate better permeability of their contents through the blood-brain barrier^[78,100-102]^. One of the R-CHOP chemotherapy medications, doxorubicin (DOX), has been investigated in multiple studies for improved delivery via EVs^[1,87,100-103]^. EV-encapsulated DOX was found to have faster uptake, more intracellular accumulation, and better potency in cell cultures than free DOX or liposomal DOX^[87]^. Another *in vitro* study showed DOX in EVs was preferentially delivered to HeLa cancer cells as compared to HEK293 cells, indicating targeted delivery to cancer cells *vs.* immortalized non-malignant cells^[103]^. With opportunities to engineer EVs for better drug delivery, R-CHOP could be optimized to improve recovery for NHL patients.

### CAR-T Therapy

EVs appear to have a biological function in the context of CAR-T therapy. CAR-T therapy begins with the removal of the NHL patient’s T cells with leukapheresis. Next, those T cells are activated, transfected or transduced with chimeric antigen receptor (CAR) genes, and expanded. These CAR-T cells are infused back to the patient after the patient completes lymphodepletion chemotherapy^[104]^. When the CAR-T cell encounters a tumor antigen matching its CAR, T cell receptor signaling with costimulatory signaling releases perforin and granzymes from the CAR-T cell. Along with the Fas ligand from the CAR-T cell binding to the Fas receptor on the tumor cell, the perforin and granzymes induce apoptosis in the tumor cell^[104]^. CAR-T therapy was initially developed to target the CD19 antigen for B-cell NHL patients^[105-107]^. CAR-T therapy clinical studies for B-cell NHL were also initiated to target the B-cell activating factor receptor (BAFF-R), CD20, CD22, CD79b, CD37, programmed cell death protein 1 (PD-1), and the human immunoglobulin kappa light chain^[107]^. Alongside this process for CAR-T therapy, EVs have been indicated as both detractors and enhancers.

The influence of EVs on CAR-T therapy has intriguing clinical implications. As CAR-T cells are prone to on-target/off-tumor effects, CD19+ EVs may negatively affect CAR-T therapy efficacy, similar to how CD20+ EVs reduce R-CHOP efficacy with rituximab^[76,85,108]^. As compared to CD19- EVs from K562 lymphoblast cells and HepG2 hepatoma cells, Zhu *et al.* found that CD19+ EVs from Nalm-6 leukemia cells induced premature cytokine release and quicker exhaustion of CAR-T cells specific for CD19, and that the CD19+ EVs reduced the anti-tumor efficacy of the CAR-T cells in NOG mice with leukemia^[88]^. Ukrainskaya *et al.* revealed that in contrast to Nalm-6 EVs with CD19 knocked out, Nalm-6 CD19+ EVs had increased binding and uptake with CD19 CAR-T cells, which had increased activation. These CD19+ EVs were positively correlated with higher levels of pro-inflammatory cytokines released and increased upregulation of activation genes. However, these CD19+ EVs were shown to accelerate CD19 CAR-T cell exhaustion. When paired with programmed death-ligand 1 (PD-L1) expression on the EVs, these CD19+/PD-L1+ EVs reduced the killing of Nalm-6 leukemia cells by the CAR-T cells^[89]^. With another subtype of NHL, chronic lymphocytic leukemia (CLL), PD-L1+ EVs were derived from the plasma of CLL patients. Increasing concentrations of these PD-L1+ EVs were demonstrated to induce more exhaustion in CD19 CAR-T cells and less killing of CD19+ JeKo-1 cells by the CD19 CAR-T cells^[90]^. This phenomenon is consistent with PD-L1+ EVs utilized for immune suppression for patients with acute graft *vs.* host disease^[109]^. Conversely, EVs with high CD19 expression from transfected HEK293T cells were found to advance activation, expansion, and maturation of CD19 CAR-T cells derived from healthy donors *in vitro*, and to elicit more anti-tumor activity through functional persistence of the CD19 CAR-T cells in mice grafted with NHL Raji cells^[91]^. This same group showed similar success upon mice grafted with NHL Raji cells, with CD19 CAR-T cells paired with EVs expressing both interleukin-12 (IL-12) and CD19 on the surface membrane^[92]^. Hence, endogenous CD19+ EVs could be viewed as detractors or enhancers of CAR-T therapy. It seems that C19+ EVs stimulate CAR-T cell response in the short term for enhanced apoptosis of cancer cells in the immediate vicinity, yet this immune response may quickly become exhausted.

CAR-T cell-associated EVs are a potential therapy for the treatment of NHL patients. T lymphocytes secrete EVs that conserve the Fas ligand as well as perforin and granzyme expression and induce apoptosis in adjacent cells^[93]^. In a couple studies, CAR-T cell-derived EVs were engineered to target epithelial growth factor receptor (EGFR) on colorectal cancer cells, human epidermal growth factor receptor 2 (HER2) on HER2+ breast cancer cells, and mesothelin (MSLN) on triple-negative breast cancer cells^[94,95]^. Both studies showed anti-tumor activity *in vitro* and in immunodeficient mice^[94,95]^. An additional study indicated HER2 CAR-T EVs increased apoptotic activity on breast cancer SKBR cells and ovarian cancer SKOV and OVCAR3 cells^[96]^. Though NHL response to CAR-T EVs was not examined in these studies, uptake of HEK293T-derived CD19 CAR EVs and their affiliated tropism for the *MYC* gene were demonstrated in NHL Raji cells^[97]^. HEK293T-derived CD19 CAR EVs also showed more cytotoxicity and pro-apoptotic genes in CD19+ leukemia B cells than CD19- cells^[98]^. Additionally, CAR-T cell-derived EVs were revealed to release substantially less cytokines than CAR-T cells in mice^[94]^. Cytokine release syndrome is a common side effect of CAR-T therapy, with a 55.3% incidence rate in hematological malignancies and can sometimes be lethal^[110]^. Though uncommon, NHL has a poor prognosis with metastasis to the brain and spinal cord meninges, a condition known as leptomeningeal metastasis (LM)^[111]^. With better permeability across the blood-brain barrier, CAR-T EVs could improve outcomes for treating NHL LM patients^[78,112]^.

## CONCLUSIONS

Due to their variety of features, EVs offer multifaceted data to harness for the treatment of NHL. NHL-associated EVs were shown to provide information as diagnostic, prognostic, and predictive biomarkers. Diagnosis can be gleaned from their miRNA, mRNA, antigen expression, or proteomic diversity. Prognosis was deduced from their miRNA and mRNA cargo, as well as their proteomes. Treatment prediction was indicated by their miRNA, mRNA, and potentially immunoglobulin expression with VDJ recombination. The influence of EVs with regard to R-CHOP and CAR-T therapy demonstrated remarkable results. CD20+ EVs deter R-CHOP efficacy, but EVs can be optimized for better R-CHOP chemotherapy delivery. CD19+ EVs stimulate CAR-T cells with so much activation, which could be dysfunctional for efficacy. However, EVs could also be engineered to express CARs and avert tumor growth with fewer side effects than CAR-T cells. These diverse elements of EVs provide utility for monitoring and treating NHL through its progression and resolution for patients.

What is most promising clinically about EVs is they can provide both qualitative and quantitative data continuously throughout the NHL disease^[113]^. Bioinformatics with genomics, RNA sequencing, and proteomics have revealed remarkable analysis for EVs and NHL^[47,113-115]^. These increasing aspects of NHL EVs supply ample information for applied data science through machine learning and artificial intelligence. With several types of numerical EV data categorized as diagnostic, prognostic, or predictive, a multidimensional array can be formatted for more refined and precise assessments of NHL status and recommendations for treatment. Additional EV data from the categories of enhancers and detractors would further enhance this computational model. Data analysis could optimize the administration of CAR-T cell-associated EVs as an adjuvant in conjunction with or in lieu of CAR-T cells^[94]^. In a feedback loop, EV data can continuously monitor and return information on NHL patient status from response to treatments via recursive algorithms for machine learning^[8,116,117]^. Artificial intelligence will not supplant the medical oncologist any time soon but can be an additional tool for guidance on making more informed decisions specific to the patient and advancing NHL precision medicine based on EVs^[8,116,117]^.

## DECLARATIONS

### Copyright

© The Author(s) 2024.

## References

[1] Xu R, Rai A, Chen M, Suwakulsiri W, Greening DW, Simpson RJ (2018). Extracellular vesicles in cancer - implications for future improvements in cancer care. Nat Rev Clin Oncol.

[2] Chang W, Cerione RA, Antonyak MA

[3] Ye Z, Chen W, Li G, Huang J, Lei J (2024). Tissue-derived extracellular vesicles in cancer progression: mechanisms, roles, and potential applications. Cancer Metastasis Rev.

[4] Crow J, Samuel G, Godwin AK (2019). Beyond tumor mutational burden: potential and limitations in using exosomes to predict response to immunotherapy. Expert Rev Mol Diagn.

[5] Trinidad CV, Tetlow AL, Bantis LE, Godwin AK (2020). Reducing ovarian cancer mortality through early detection: approaches using circulating biomarkers. Cancer Prev Res.

[6] Hinestrosa JP, Kurzrock R, Lewis JM (2022). Early-stage multi-cancer detection using an extracellular vesicle protein-based blood test. Commun Med.

[7] Irmer B, Chandrabalan S, Maas L, Bleckmann A, Menck K (2023). Extracellular vesicles in liquid biopsies as biomarkers for solid tumors. Cancers.

[8] Yu D, Li Y, Wang M (2022). Exosomes as a new frontier of cancer liquid biopsy. Mol Cancer.

[9] Zhou E, Li Y, Wu F (2021). Circulating extracellular vesicles are effective biomarkers for predicting response to cancer therapy. EBioMedicine.

[10] Rayamajhi S, Sipes J, Tetlow AL, Saha S, Bansal A, Godwin AK (2024). Extracellular vesicles as liquid biopsy biomarkers across the cancer journey: from early detection to recurrence. Clin Chem.

[11] Cheng S, Li Y, Yan H (2021). Advances in microfluidic extracellular vesicle analysis for cancer diagnostics. Lab Chip.

[12] Théry C, Witwer KW, Aikawa E (2018). Minimal information for studies of extracellular vesicles 2018 (MISEV2018): a position statement of the international society for extracellular vesicles and update of the MISEV2014 guidelines. J Extracell Vesicles.

[13] Jeppesen DK, Zhang Q, Franklin JL, Coffey RJ (2023). Extracellular vesicles and nanoparticles: emerging complexities. Trends Cell Biol.

[14] Nowak M, Górczyńska J, Kołodzińska K, Rubin J, Choromańska A (2023). Extracellular vesicles as drug transporters. Int J Mol Sci.

[15] Hanahan D, Weinberg RA (2011). Hallmarks of cancer: the next generation. Cell.

[16] (2018). van Niel G, D'Angelo G, Raposo G. Shedding light on the cell biology of extracellular vesicles. Nat Rev Mol Cell Biol.

[17] Decruyenaere P, Offner F, Vandesompele J (2021). Circulating RNA biomarkers in diffuse large B-cell lymphoma: a systematic review. Exp Hematol Oncol.

[18] Ghanam J, Chetty VK, Barthel L, Reinhardt D, Hoyer PF, Thakur BK (2022). DNA in extracellular vesicles: from evolution to its current application in health and disease. Cell Biosci.

[19] Malkin EZ, Bratman SV (2020). Bioactive DNA from extracellular vesicles and particles. Cell Death Dis.

[20] Elzanowska J, Semira C, Costa-Silva B (2021). DNA in extracellular vesicles: biological and clinical aspects. Mol Oncol.

[21] Jabalee J, Towle R, Garnis C (2018). The role of extracellular vesicles in cancer: cargo, function, and therapeutic implications. Cells.

[22] Kikuchi S, Yoshioka Y, Prieto-Vila M, Ochiya T (2019). Involvement of extracellular vesicles in vascular-related functions in cancer progression and metastasis. Int J Mol Sci.

[23] Bhatta B, Luz I, Krueger C (2021). Cancer cells shuttle extracellular vesicles containing oncogenic mutant p53 proteins to the tumor microenvironment. Cancers (Basel).

[24] Putz U, Mah S, Goh CP, Low LH, Howitt J, Tan SS (2015). PTEN secretion in exosomes. Methods.

[25] Buzas EI (2023). The roles of extracellular vesicles in the immune system. Nat Rev Immunol.

[26] Al-Mayah AH, Bright SJ, Bowler DA, Slijepcevic P, Goodwin E, Kadhim MA (2017). Exosome-mediated telomere instability in human breast epithelial cancer cells after X irradiation. Radiat Res.

[27] Wang Z, Lieberman PM (2016). The crosstalk of telomere dysfunction and inflammation through cell-free TERRA containing exosomes. RNA Biol.

[28] Terlecki-Zaniewicz L, Lämmermann I, Latreille J (2018). Small extracellular vesicles and their miRNA cargo are anti-apoptotic members of the senescence-associated secretory phenotype. Aging.

[29] Wang J, Hendrix A, Hernot S (2014). Bone marrow stromal cell-derived exosomes as communicators in drug resistance in multiple myeloma cells. Blood.

[30] Yang L, Wu XH, Wang D, Luo CL, Chen LX (2013). Bladder cancer cell-derived exosomes inhibit tumor cell apoptosis and induce cell proliferation in vitro. Mol Med Rep.

[31] Federici C, Petrucci F, Caimi S (2014). Exosome release and low pH belong to a framework of resistance of human melanoma cells to cisplatin. PLoS One.

[32] Ciravolo V, Huber V, Ghedini GC (2012). Potential role of HER2-overexpressing exosomes in countering trastuzumab-based therapy. J Cell Physiol.

[33] Samuel P, Mulcahy LA, Furlong F (2018). Cisplatin induces the release of extracellular vesicles from ovarian cancer cells that can induce invasiveness and drug resistance in bystander cells. Philos Trans R Soc Lond B Biol Sci.

[34] Ferlizza E, Romaniello D, Borrelli F (2023). Extracellular vesicles and epidermal growth factor receptor activation: interplay of drivers in cancer progression. Cancers (Basel).

[35] Jacobson CA, Longo DL

[36] https://www.cancer.gov/types/lymphoma/hp/adult-nhl-treatment-pdq.

[37] Parham P

[38] https://www.mountsinai.org/health-library/report/non-hodgkins-lymphoma.

[39] https://kumc.hosted.panopto.com/Panopto/Pages/Embed.aspx?pid=54880ca6-5c1e-431f-80a0-ac8d0115fce4&id=b923d9a8-a3de-4f68-b15e-ab3c0164b485.

[40] Thandra KC, Barsouk A, Saginala K, Padala SA, Barsouk A, Rawla P (2021). Epidemiology of non-Hodgkin’s lymphoma. Med Sci.

[41] Navarro-Tableros V, Gomez Y, Camussi G, Brizzi MF (2018). Extracellular vesicles: new players in lymphomas. Int J Mol Sci.

[42] Chu Y, Liu Y, Fang X (2023). The epidemiological patterns of non-Hodgkin lymphoma: global estimates of disease burden, risk factors, and temporal trends. Front Oncol.

[43] https://seer.cancer.gov/statfacts/html/nhl.html.

[44] Padala SA, Kallam A https://www.ncbi.nlm.nih.gov/books/NBK557796.

[45] Basso K, Dalla-Favera R (2015). Germinal centres and B cell lymphomagenesis. Nat Rev Immunol.

[46] Wagener R, Lopez C, Siebert R

[47] Schmitz R, Wright GW, Huang DW (2018). Genetics and pathogenesis of diffuse large B-cell lymphoma. N Engl J Med.

[48] Ofori K, Bhagat G, Rai AJ (2021). Exosomes and extracellular vesicles as liquid biopsy biomarkers in diffuse large B-cell lymphoma: current state of the art and unmet clinical needs. Br J Clin Pharmacol.

[49] Wang L, Li LR, Young KH (2020). New agents and regimens for diffuse large B cell lymphoma. J Hematol Oncol.

[50] https://www.cancer.gov/news-events/cancer-currents-blog/2022/nhl-car-t-cells-belinda-transform-zuma7.

[51] Lai CP, Mardini O, Ericsson M (2014). Dynamic biodistribution of extracellular vesicles in vivo using a multimodal imaging reporter. ACS Nano.

[52] Ciferri MC, Quarto R, Tasso R (2021). Extracellular vesicles as biomarkers and therapeutic tools: from pre-clinical to clinical applications. Biology.

[53] Razvi E (2014). Prognostic vs. predictive biomarkers. Clinical OMICs.

[54] Oldenhuis CN, Oosting SF, Gietema JA, de Vries EG (2008). Prognostic versus predictive value of biomarkers in oncology. Eur J Cancer.

[55] Gutknecht MF, Holodick NE, Rothstein TL (2023). B cell extracellular vesicles contain monomeric IgM that binds antigen and enters target cells. iScience.

[56] Zheng D, Huo M, Li B (2020). The role of exosomes and exosomal microRNA in cardiovascular disease. Front Cell Dev Biol.

[57] O'Brien K, Breyne K, Ughetto S, Laurent LC, Breakefield XO (2020). RNA delivery by extracellular vesicles in mammalian cells and its applications. Nat Rev Mol Cell Biol.

[58] Yu W, Hurley J, Roberts D (2021). Exosome-based liquid biopsies in cancer: opportunities and challenges. Ann Oncol.

[59] Wei Z, Batagov AO, Schinelli S (2017). Coding and noncoding landscape of extracellular RNA released by human glioma stem cells. Nat Commun.

[60] Lai CP, Kim EY, Badr CE (2015). Visualization and tracking of tumour extracellular vesicle delivery and RNA translation using multiplexed reporters. Nat Commun.

[61] Cao D, Cao X, Jiang Y (2022). Circulating exosomal microRNAs as diagnostic and prognostic biomarkers in patients with diffuse large B-cell lymphoma. Hematol Oncol.

[62] Liu J, Han Y, Hu S (2021). Circulating exosomal MiR-107 restrains tumorigenesis in diffuse large B-cell lymphoma by targeting 14-3-3η. Front Cell Dev Biol.

[63] Xiao XB, Gu Y, Sun DL (2019). Effect of rituximab combined with chemotherapy on the expression of serum exosome miR-451a in patients with diffuse large b-cell lymphoma. Eur Rev Med Pharmacol Sci.

[64] Feng Y, Zhong M, Zeng S (2019). Exosome-derived miRNAs as predictive biomarkers for diffuse large B-cell lymphoma chemotherapy resistance. Epigenomics.

[65] Zare N, Haghjooy Javanmard S, Mehrzad V, Eskandari N, Kefayat A (2019). Evaluation of exosomal miR-155, let-7g and let-7i levels as a potential noninvasive biomarker among refractory/relapsed patients, responsive patients and patients receiving R-CHOP. Leuk Lymphoma.

[66] Yazdanparast S, Huang Z, Keramat S (2022). The roles of exosomal microRNAs in diffuse large B-cell lymphoma: diagnosis, prognosis, clinical application, and biomolecular mechanisms. Front Oncol.

[67] Provencio M, Rodríguez M, Cantos B (2017). mRNA in exosomas as a liquid biopsy in non-Hodgkin lymphoma: a multicentric study by the Spanish lymphoma oncology group. Oncotarget.

[68] Bang YH, Shim JH, Ryu KJ (2022). Clinical relevance of serum-derived exosomal messenger RNA sequencing in patients with non-Hodgkin lymphoma. J Cancer.

[69] Rutherford SC, Fachel AA, Li S (2018). Extracellular vesicles in DLBCL provide abundant clues to aberrant transcriptional programming and genomic alterations. Blood.

[70] Chapuy B, Stewart C, Dunford AJ (2018). Molecular subtypes of diffuse large B cell lymphoma are associated with distinct pathogenic mechanisms and outcomes. Nat Med.

[71] Hoshino A, Kim HS, Bojmar L (2020). Extracellular vesicle and particle biomarkers define multiple human cancers. Cell.

[72] Liu H, Lu J, Dong Z (2018). Cell-of-Origin subtype prediction of diffuse large B-cell lymphoma using gene expression and proteomic data. Blood.

[73] Bram Ednersson S, Stern M, Fagman H (2019). Quantitative proteomics in diffuse large B-cell lymphoma patients reveal novel overexpressed proteins and potentially druggable targets in the ABC subtype. Blood.

[74] Carvalho AS, Baeta H, Henriques AFA (2021). Proteomic landscape of extracellular vesicles for diffuse large B-cell lymphoma subtyping. Int J Mol Sci.

[75] Matthiesen R, Gameiro P, Henriques A (2022). Extracellular vesicles in diffuse large B cell lymphoma: characterization and diagnostic potential. Int J Mol Sci.

[76] Oksvold MP, Kullmann A, Forfang L (2014). Expression of B-cell surface antigens in subpopulations of exosomes released from B-cell lymphoma cells. Clin Ther.

[77] Yao Y, Wei W, Sun J (2015). Proteomic analysis of exosomes derived from human lymphoma cells. Eur J Med Res.

[78] Cheng L, Hill AF (2022). Therapeutically harnessing extracellular vesicles. Nat Rev Drug Discov.

[79] Caivano A, Laurenzana I, De Luca L (2015). High serum levels of extracellular vesicles expressing malignancy-related markers are released in patients with various types of hematological neoplastic disorders. Tumour Biol.

[80] Cui M, Huang J, Zhang S, Liu Q, Liao Q, Qiu X (2021). Immunoglobulin expression in cancer cells and its critical roles in tumorigenesis. Front Immunol.

[81] Kurtz DM, Green MR, Bratman SV (2015). Noninvasive monitoring of diffuse large B-cell lymphoma by immunoglobulin high-throughput sequencing. Blood.

[82] Merryman RW, Redd RA, Taranto E (2023). Minimal residual disease in patients with diffuse large B-cell lymphoma undergoing autologous stem cell transplantation. Blood Adv.

[83] Khodadoust MS, Olsson N, Chen B (2019). B-cell lymphomas present immunoglobulin neoantigens. Blood.

[84] Couto N, Elzanowska J, Maia J (2022). IgG^+^ extracellular vesicles measure therapeutic response in advanced pancreatic cancer. Cells.

[85] Aung T, Chapuy B, Vogel D (2011). Exosomal evasion of humoral immunotherapy in aggressive B-cell lymphoma modulated by ATP-binding cassette transporter A3. Proc Natl Acad Sci U S A.

[86] Melo Garcia L, Barabé F (2021). Harnessing macrophages through the blockage of CD47: implications for acute myeloid leukemia. Cancers.

[87] Schindler C, Collinson A, Matthews C (2019). Exosomal delivery of doxorubicin enables rapid cell entry and enhanced in vitro potency. PLoS One.

[88] Zhu X, Hu H, Xiao Y (2022). Tumor-derived extracellular vesicles induce invalid cytokine release and exhaustion of CD19 CAR-T Cells. Cancer Lett.

[89] Ukrainskaya VM, Musatova OE, Volkov DV (2023). CAR-tropic extracellular vesicles carry tumor-associated antigens and modulate CAR T cell functionality. Sci Rep.

[90] Cox MJ, Lucien F, Sakemura R (2021). Leukemic extracellular vesicles induce chimeric antigen receptor T cell dysfunction in chronic lymphocytic leukemia. Mol Ther.

[91] Zhang Y, Ge T, Huang M (2023). Extracellular vesicles expressing CD19 antigen improve expansion and efficacy of CD19-targeted CAR-T cells. Int J Nanomedicine.

[92] Huang M, Zhao L, Li D, Huang L, Wang J, Zhang Y (2023). Targeted delivery of IL-12 via CD19-modified extracellular vesicles enhances CAR-T cell efficacy against lymphoma. Blood.

[93] Calvo V, Izquierdo M (2022). T lymphocyte and CAR-T cell-derived extracellular vesicles and their applications in cancer therapy. Cells.

[94] Fu W, Lei C, Liu S (2019). CAR exosomes derived from effector CAR-T cells have potent antitumour effects and low toxicity. Nat Commun.

[95] Yang P, Cao X, Cai H (2021). The exosomes derived from CAR-T cell efficiently target mesothelin and reduce triple-negative breast cancer growth. Cell Immunol.

[96] Aharon A, Horn G, Bar-Lev TH (2021). Extracellular vesicles derived from chimeric antigen receptor-t cells: a potential therapy for cancer. Hum Gene Ther.

[97] Xu Q, Zhang Z, Zhao L (2020). Tropism-facilitated delivery of CRISPR/Cas9 system with chimeric antigen receptor-extracellular vesicles against B-cell malignancies. J Control Release.

[98] Haque S, Vaiselbuh SR (2021). CD19 chimeric antigen receptor-exosome targets CD19 positive B-lineage acute lymphocytic leukemia and induces cytotoxicity. Cancers.

[99] Marleau AM, Chen CS, Joyce JA, Tullis RH (2012). Exosome removal as a therapeutic adjuvant in cancer. J Transl Med.

[100] Luan X, Sansanaphongpricha K, Myers I, Chen H, Yuan H, Sun D (2017). Engineering exosomes as refined biological nanoplatforms for drug delivery. Acta Pharmacol Sin.

[101] Ferreira D, Moreira JN, Rodrigues LR (2022). New advances in exosome-based targeted drug delivery systems. Crit Rev Oncol Hematol.

[102] Pirisinu M, Pham TC, Zhang DX, Hong TN, Nguyen LT, Le MT (2022). Extracellular vesicles as natural therapeutic agents and innate drug delivery systems for cancer treatment: recent advances, current obstacles, and challenges for clinical translation. Semin Cancer Biol.

[103] Goh WJ, Lee CK, Zou S, Woon EC, Czarny B, Pastorin G (2017). Doxorubicin-loaded cell-derived nanovesicles: an alternative targeted approach for anti-tumor therapy. Int J Nanomedicine.

[104] Makita S, Yoshimura K, Tobinai K (2017). Clinical development of anti-CD19 chimeric antigen receptor T-cell therapy for B-cell non-Hodgkin lymphoma. Cancer Sci.

[105] Neelapu SS, Locke FL, Bartlett NL (2017). Axicabtagene ciloleucel CAR T-cell therapy in refractory large B-cell lymphoma. N Engl J Med.

[106] Schuster SJ, Bishop MR, Tam CS, JULIET Investigators (2019). Tisagenlecleucel in adult relapsed or refractory diffuse large B-cell lymphoma. N Engl J Med.

[107] Yin Z, Zhang Y, Wang X (2021). Advances in chimeric antigen receptor T-cell therapy for B-cell non-Hodgkin lymphoma. Biomark Res.

[108] Sterner RC, Sterner RM (2021). CAR-T cell therapy: current limitations and potential strategies. Blood Cancer J.

[109] Li M, Soder R, Abhyankar S (2021). WJMSC-derived small extracellular vesicle enhance T cell suppression through PD-L1. J Extracell Vesicles.

[110] Miao L, Zhang Z, Ren Z, Li Y (2021). Reactions related to CAR-T cell therapy. Front Immunol.

[111] Meng X, Yu J, Fan Q (2018). Characteristics and outcomes of non-Hodgkin's lymphoma patients with leptomeningeal metastases. Int J Clin Oncol.

[112] Tang XJ, Sun XY, Huang KM (2015). Therapeutic potential of CAR-T cell-derived exosomes: a cell-free modality for targeted cancer therapy. Oncotarget.

[113] Hendrix A, Lippens L, Pinheiro C (2023). Extracellular vesicle analysis. Nat Rev Methods Primers.

[114] Steen CB, Luca BA, Esfahani MS (2021). The landscape of tumor cell states and ecosystems in diffuse large B cell lymphoma. Cancer Cell.

[115] Bram Ednersson S, Stern M, Fagman H (2021). Proteomic analysis in diffuse large B-cell lymphoma identifies dysregulated tumor microenvironment proteins in non-GCB/ABC subtype patients. Leuk Lymphoma.

[116] Topol EJ (2019). High-performance medicine: the convergence of human and artificial intelligence. Nat Med.

[117] Rajpurkar P, Chen E, Banerjee O, Topol EJ (2022). AI in health and medicine. Nat Med.

